# An Unusual Case of Non-traumatic Tension Pneumocephalus

**DOI:** 10.7759/cureus.83064

**Published:** 2025-04-27

**Authors:** Sumana Kedilaya, Niharika Gupta, Hejjaji Anand Krishnamurthy, Arjun Raju P, Deepak Haldipur

**Affiliations:** 1 Radiology, Tenet Diagnostics, Bangalore, IND; 2 Ear, Nose, and Throat, Trustwell Hospitals, Bangalore, IND

**Keywords:** csf fistula, csf rhinorrhea, mri cisternogram, sphenoid encephalocele, spontaneous tension pneumocephalus

## Abstract

Tension pneumocephalus is a life-threatening situation in neurosurgery. It can occur due to various causes, such as trauma, complications of neurosurgery or sinus surgery, and also in conditions where there is a CSF leak. We present an unusual case of a 53-year-old woman who came with complaints of severe headache and CSF rhinorrhea. On radiologic evaluation, tension pneumocephalus was noted as a result of sphenoid encephalocele. Sphenoidal encephaloceles are herniations of brain parenchyma, meninges, and CSF through a defect in the sphenoid bone. It typically presents as CSF rhinorrhea. Rarely, tension pneumocephalus can occur in the form of a severe headache. The recognition of clinical and radiologic features can help in making an accurate diagnosis of this rare condition at the earliest.

## Introduction

Pneumocephalus refers to the presence of intracranial air. It is mostly asymptomatic. The most common causes are head trauma and iatrogenic causes such as neurosurgery, sinus surgery, or epidural injections [1]. Non-traumatic causes are relatively uncommon and occur due to CSF leak, pneumosinus dilatans, or excessive vigorous sneezing [2].

Tension pneumocephalus refers to the accumulation of air within the cranium, causing elevated intracranial pressure, presenting with headache, vomiting, and neurological deficits. It can lead to coma and become a life-threatening condition [3].

Sphenoid encephalocele is a herniation of the temporal lobe through a defect within the sphenoid bone. It is accompanied by a leak of CSF. Very rarely, tension pneumocephalus can occur in association with sphenoid encephalocele and pose as a neurosurgical emergency [4]. In this report, we describe a case of spontaneous tension pneumocephalus secondary to sphenoid encephalocele in a 53-year-old woman.

## Case presentation

A 53-year-old lady presented to the clinic with a history of watery discharge from the left nostril for three weeks. She also had episodes of on-and-off severe headaches in the last three weeks. There was no history of previous trauma, sinus surgery, neurosurgery, meningitis, or intracranial tumors. No similar episodes had occurred in the past. On clinical examination, clear watery discharge was seen coming from the left nostril. There were no clinical signs of meningitis or elevated intracranial pressure, such as papilledema. CSF rhinorrhea was suspected, and she was subsequently referred to our diagnostic center for a CT scan of the paranasal sinuses and brain.

**Figure 1:**
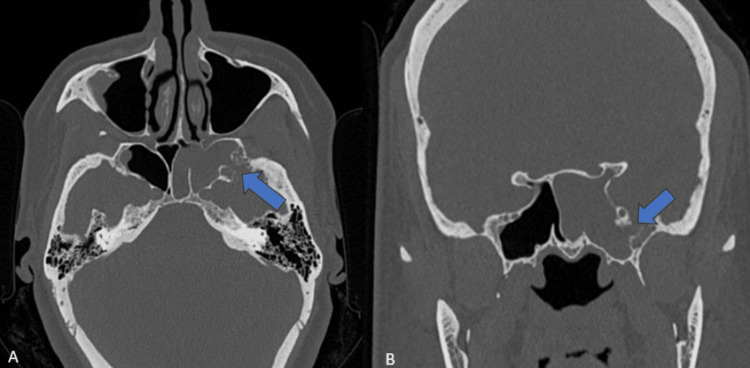
Axial and coronal CT sections at the level of the sphenoid sinus A: axial CT section; B: coronal CT section shows a focal defect (blue arrow) in the left lateral recess of the sphenoid sinus. There is complete opacification of the left side of the sphenoid sinus with fluid.

CT revealed a bony defect in the left lateral recess of the sphenoid sinus measuring 0.6 x 0.4cm, as can be seen in Figure 1. The left half of the sphenoid sinus was completely opacified by fluid (Figure 1). Small focal areas of bony scalloping were seen in the region of the left lateral recess of the sphenoid sinus.

**Figure 2:**
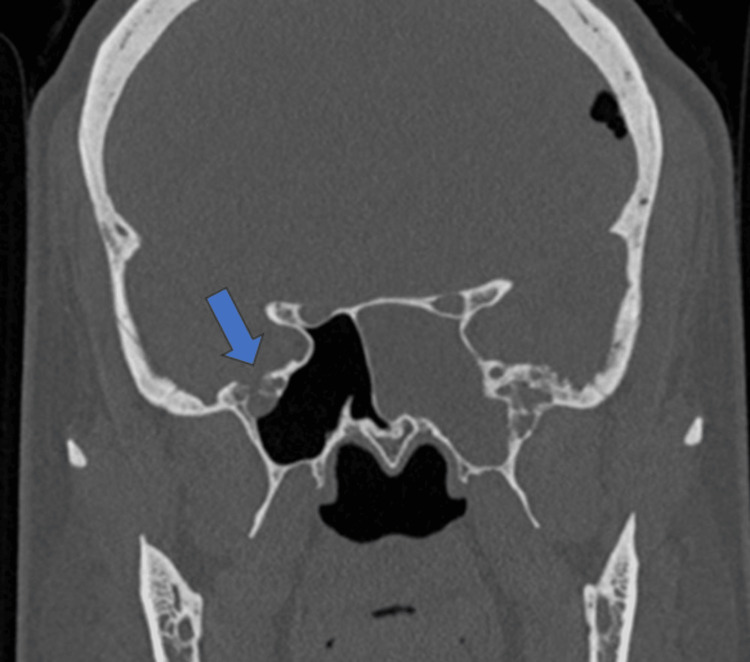
Coronal CT section at the level of sphenoid sinuses The image shows a small focal defect (blue arrow) in the right lateral recess of the sphenoid sinus with mild adjacent fluid opacification.

A small focal defect was also seen in the right lateral recess of the sphenoid sinus, as can be seen in Figure 2. However, the right half of the sphenoid sinus was clear with no fluid opacification.

**Figure 3:**
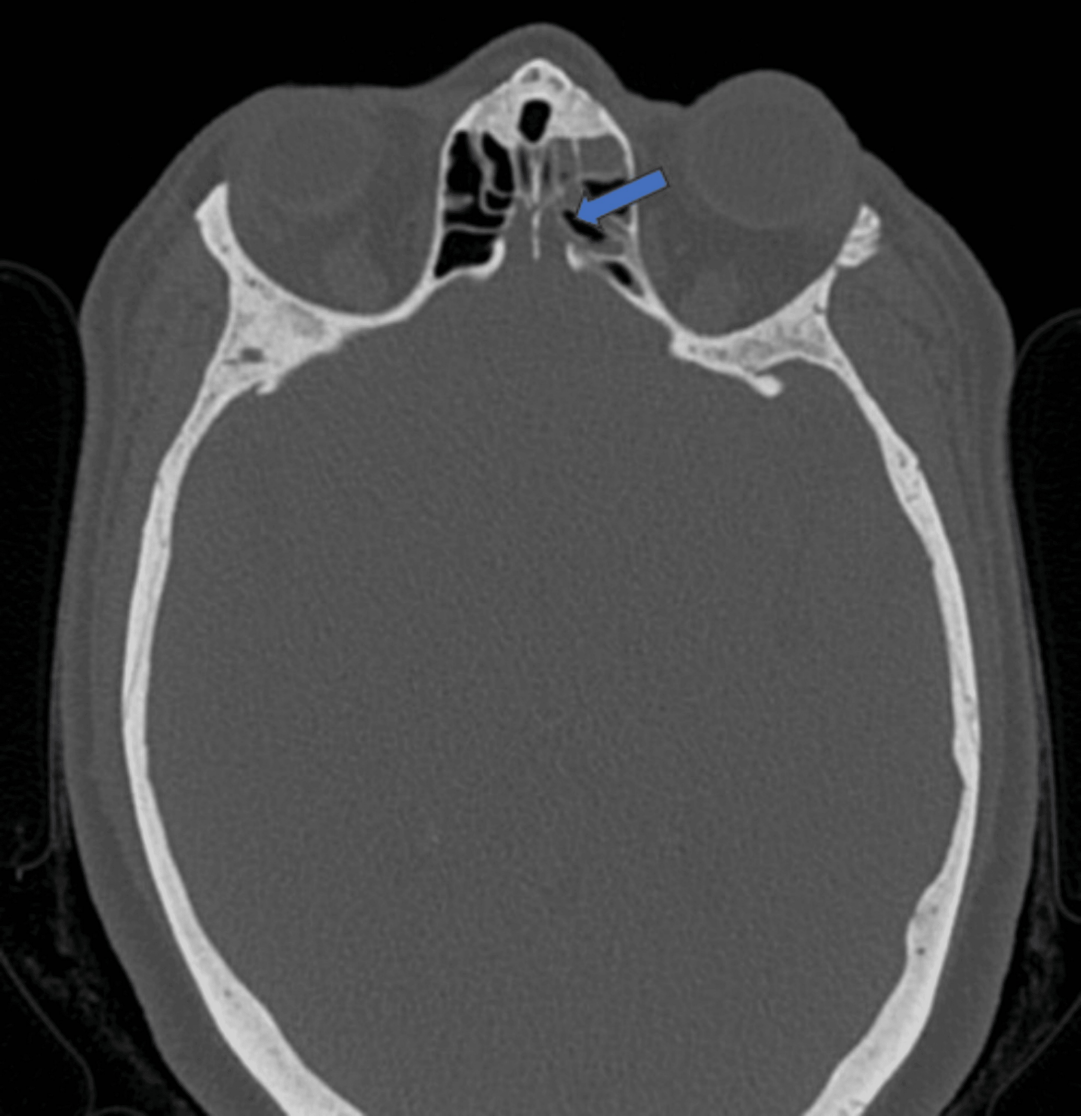
Axial section of CT paranasal sinus at the level of cribriform plate The image shows a focal dehiscence (blue arrow) in the left cribriform plate.

Focal dehiscence of the left cribriform plate was also appreciated (Figure 3).

**Figure 4:**
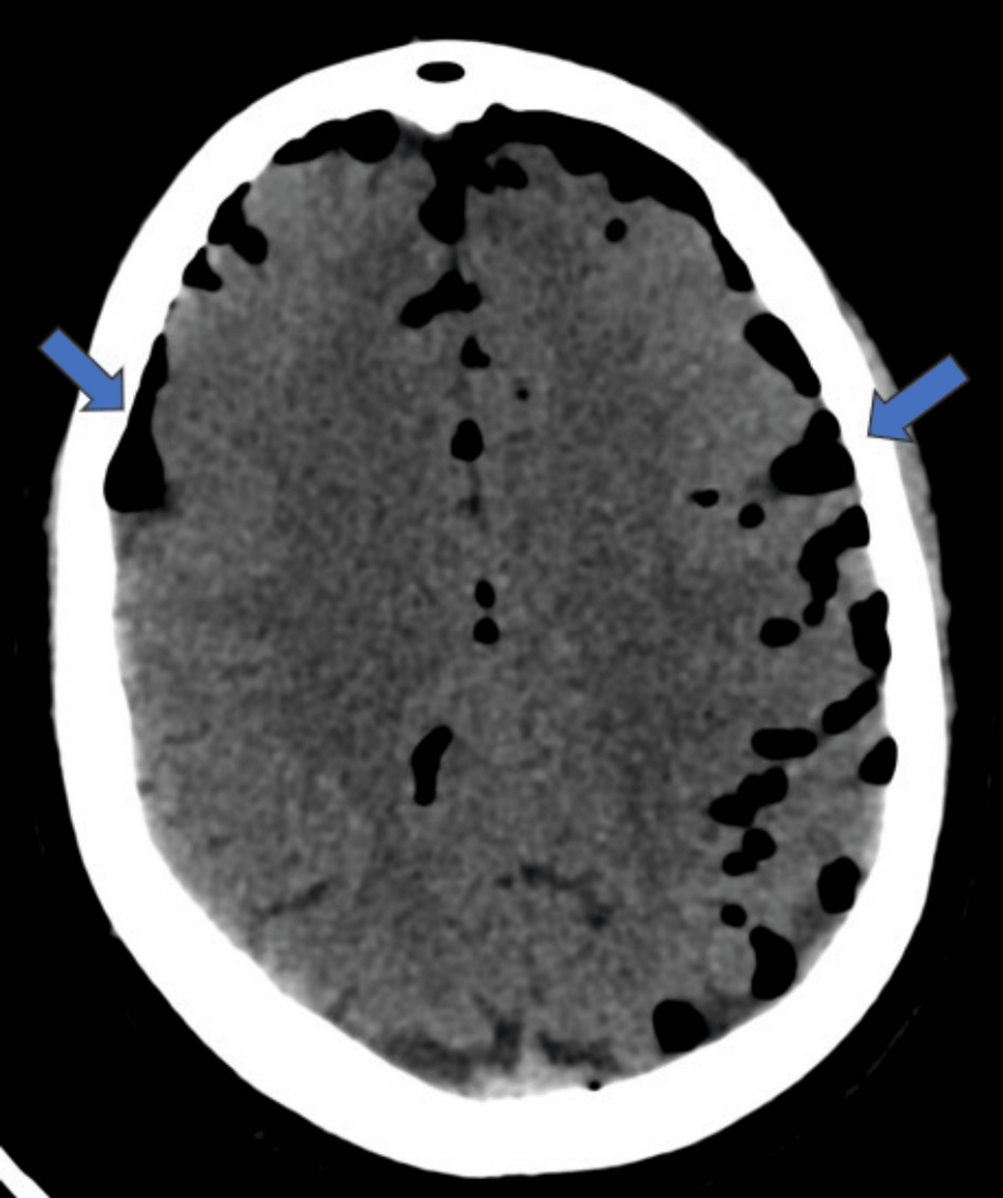
Axial CT brain at the level of frontoparietal lobes The image shows extensive air foci (blue arrows) along the subarachnoid and subdural spaces of bilateral cerebral convexities, predominantly on the left side.

In the brain sections, extensive air foci were seen along the subarachnoid spaces of bilateral fronto-parietal sulci, bilateral Sylvian fissures, perimesencephalic cisterns, prepontine cistern, and cerebellar folia (Figure 4). Similar air foci were seen along the subdural spaces of bilateral anterior frontal and anterior temporal convexities.

Following this, a suspicion of sphenoid encephalocele was raised, and the patient was subjected to an MRI cisternogram to confirm the same.

**Figure 5:**
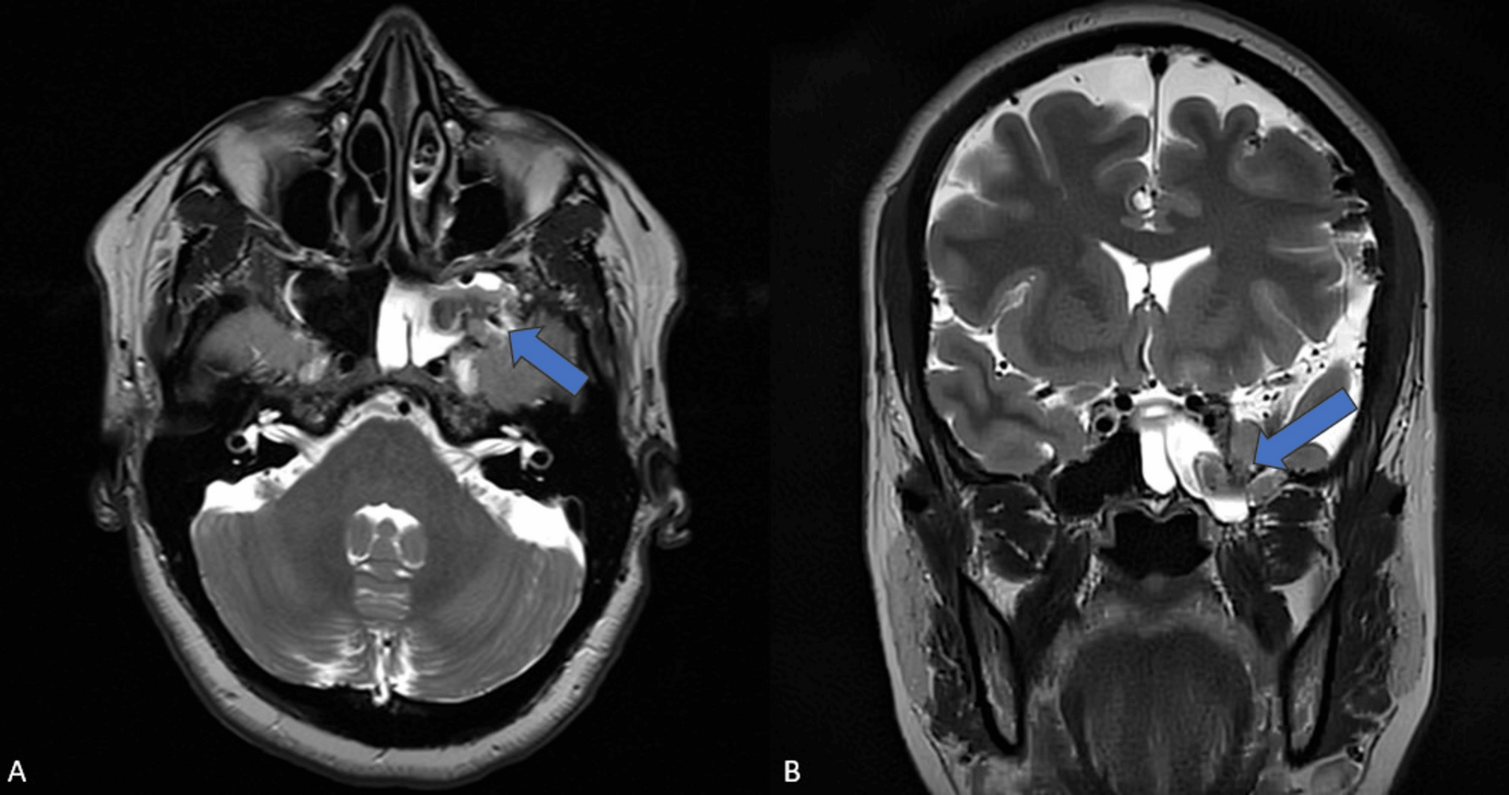
MRI brain axial and coronal T2W image at the level of the sphenoid sinus A: axial T2W MRI brain; B: coronal T2W MRI brain shows a defect (blue arrow) in the left lateral recess of the sphenoid sinus with herniation of meninges, CSF, and a portion of the left anterior temporal lobe.

**Figure 6:**
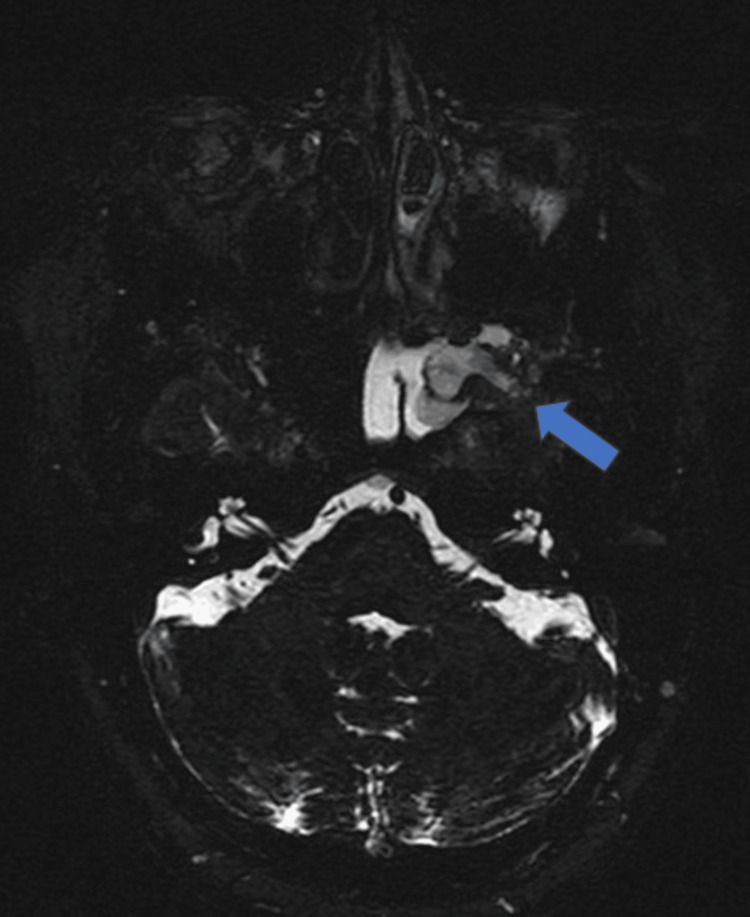
Axial heavily T2W CISS image (cisternogram) at the level of the sphenoid sinus MR cisternogram shows clear delineation of defect (blue arrow) in left lateral recess of sphenoid sinus with protrusion of meninges, CSF and portion of left anterior temporal lobe. The left half of sphenoid sinus is completely filled with CSF. CISS: constructive interference in steady state

MRI also revealed a bony defect in the left lateral recess of the sphenoid sinus with herniation of a portion of the left anteromedial temporal lobe, dura, and CSF into the sphenoid sinus (Figures 5, 6). The left half of the sphenoid sinus was completely filled with CSF.

**Figure 7:**
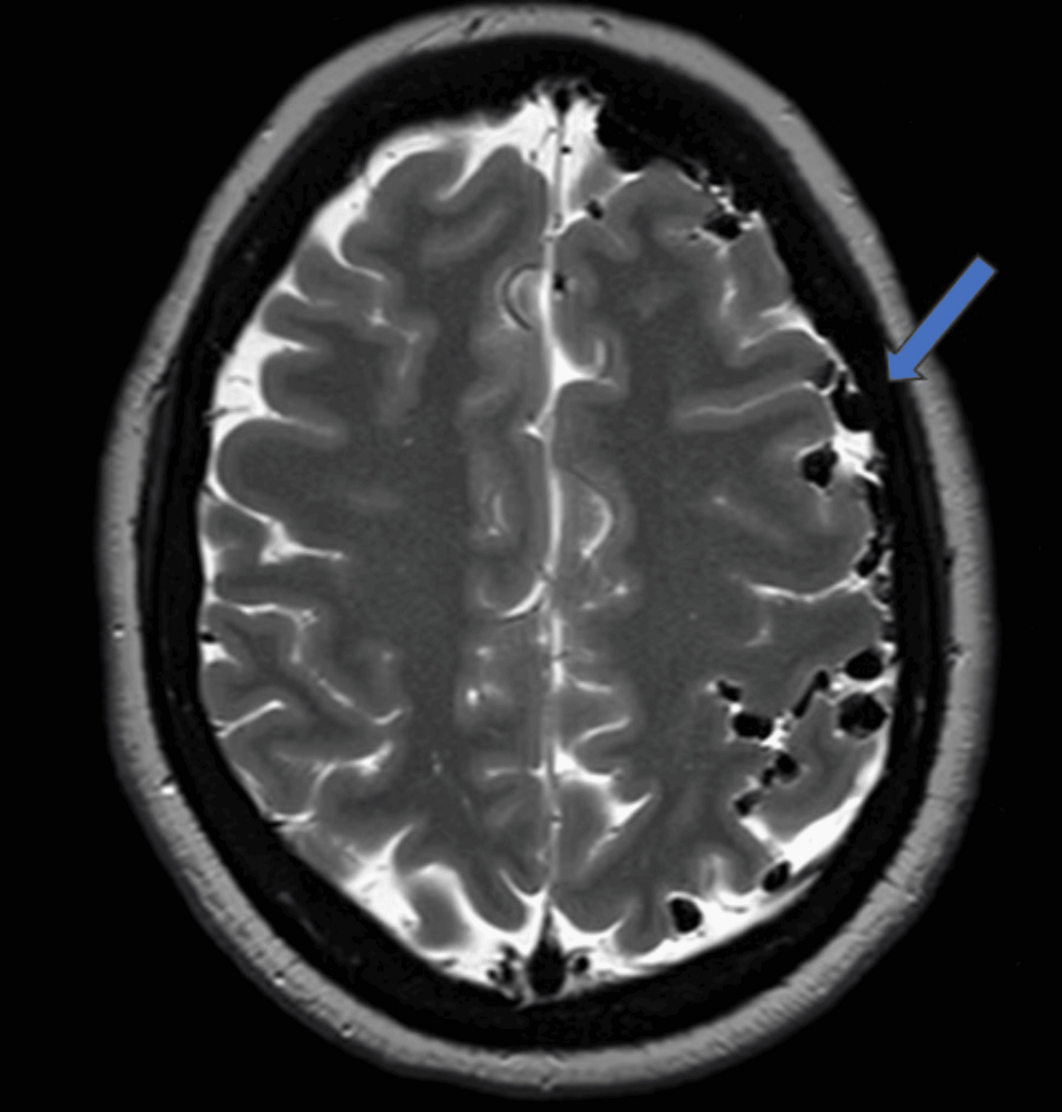
MRI brain axial T2W image at the level of the frontoparietal lobes The image shows extensive air foci (blue arrow) along the subarachnoid spaces of the brain, more on the left side.

Also noted were extensive air foci along the subarachnoid, subdural spaces, and cisterns of the brain (Figure 7).

**Figure 8:**
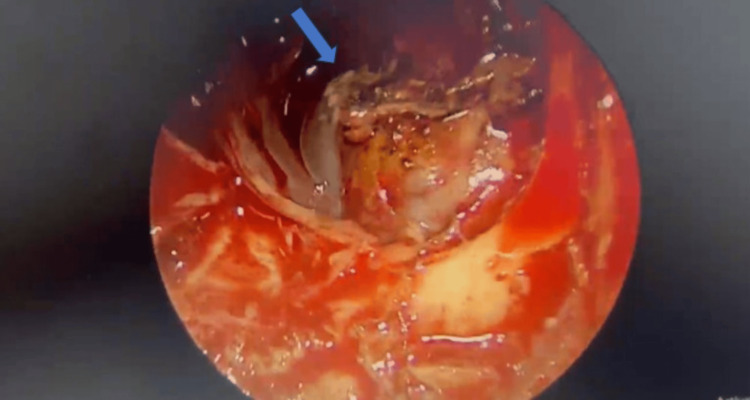
Intra-operative image The image shows the herniated portion of the temporal lobe (blue arrow) through the defect in the left lateral recess of the sphenoid sinus.

We arrived at the diagnosis of left sphenoidal encephalocele with resultant tension pneumocephalus. The patient was then referred to a head and neck surgeon. She underwent endoscopic endonasal CSF leak repair. Per operatively, the imaging findings were confirmed (Figure 8). A thigh muscle graft was used to cover the left lateral recess bone defect. The right lateral recess defect and cribriform plate dehiscence were also repaired. Postoperatively, the patient recovered well with resolution of headache and CSF rhinorrhea.

## Discussion

Tension pneumocephalus is thought to occur in conditions where there is an intracranial dural defect causing subsequent air expansion as a result of a ball-valve mechanism [5]. It leads to elevated intracranial pressure and deterioration in consciousness and neurologic deficits and can also result in cardiac arrest [6].

Sphenoid encephaloceles rarely cause tension pneumocephalus [7]. Rupture of sphenoid encephalocele, leading to a ball-valve mechanism, can lead to air entry into the subdural and subarachnoid spaces of the cranium [4]. Very rarely, intraventricular pneumocephalus can also be seen [4]. 

CSF fistulas are leaks of CSF from the subarachnoid space of the intracranial cavity through an osteodural defect into a pneumatized structure at the base of the skull [8]. CSF fistulas are broadly divided into acquired or congenital fistulas. They can also be classified as traumatic, non-traumatic, or spontaneous fistulas. The most common cause of CSF leak is trauma, such as fracture of the skull base, or iatrogenic reasons. Non-traumatic fistulas occur as a result of tumors and infections. Elevated intracranial pressure can lead to a spontaneous CSF leak. They tend to occur in areas where the bone is weak, such as pneumatized sinuses, especially the lateral recess of the sphenoid sinus and the cribriform plate. Other factors that can increase the chance of spontaneous leaks are obesity, age, chronic inflammation, bone remodeling, and anatomical variants in the bones of the skull base. Skull base encephaloceles usually co-exist in such situations. Cephaloceles are herniations of intracranial contents via a bony defect, mostly in the sphenoid sinus or cribriform plate. Meningoceles refer to the herniation of only meninges and CSF. Encephaloceles or meningoencephaloceles are protrusions of CSF, meninges, and portions of neuroparenchyma. Skull base encephaloceles can be trans-ethmoidal, spheno-ethmoidal, spheno-orbital, and sphenoidal types [9].

Sphenoidal can be further classified as medial perisellar and lateral sphenoid encephaloceles. Medial perisellar transsphenoidal encephaloceles occur through a defect in the antero-inferior wall of the sphenoid sinus and communicate directly with the sella. Lateral sphenoid encephaloceles occur when there is a defect in the lateral recess of the sphenoid sinus through which the anterior temporal lobe protrudes [10]. Lateral sphenoid encephaloceles are one of the rarest types of skull base encephaloceles [11].

They often present with CSF rhinorrhea, which is a clear discharge through the nostril. Other presentations include symptoms of increased intracranial pressure, meningitis, and seizures. Seizures occur if the herniated brain parenchyma becomes gliotic [12].

Radiologic evaluation of sphenoid encephaloceles involves CT and MRI. CT of paranasal sinuses and brain helps in detecting bony defects in the walls of the sphenoid sinus. It also helps in assessing anatomical variations and the dehiscence of skull base bones. CT sections of the brain can detect pneumocephalus. MRI helps in visualizing the soft tissue details. Frank herniation of neuroparenchyma through bony defects is well made out on MRI. The presence of gliosis within the herniated brain can be made out. It helps in classifying the type of skull base encephalocele. It can also detect complications like features of meningitis or signs of increased intracranial pressure. Cisternograms are novel techniques for evaluating the presence of CSF leaks. CT cisternography is a minimally invasive method wherein contrast is injected intrathecally via lumbar puncture. Subsequently, CT images are acquired to see the intracranial CSF spaces and detect active CSF leaks [13].

MR cisternography, on the other hand, is a non-invasive method of appreciating CSF leak by obtaining heavily weighted T2W fat-saturated images such as 3D constructive interference steady state sequence (CISS) or 3D T2 driven equilibrium (DRIVE) sequence [14].

It can be performed without the administration of contrast. Hence, people with adverse reactions or contraindications to contrast can undergo MRI cisternograms.

The most effective way of repairing lateral sphenoidal encephaloceles is via an endoscopic endonasal approach. It is also a less invasive technique compared to the conventional transcranial approach [15].

## Conclusions

Tension pneumocephalus is a rare complication of sphenoidal encephalocele. It is important to detect tension pneumocephalus and the cause of it, since it is a life-threatening condition and requires prompt intervention. Accurate and timely diagnosis needs the use of appropriate radiologic investigations and correlating it with clinical information. MRI cisternography is a novel technique in detecting CSF leaks and encephaloceles. Surgical repair of sphenoid encephalocele includes an endoscopic endonasal approach.
